# Monetary Valuation of a Quality-Adjusted Life Year (QALY) for Depressive Disorders Among Patients and Non-Patient Respondents: A Matched Willingness to Pay Study

**DOI:** 10.32872/cpe.3855

**Published:** 2021-12-23

**Authors:** Laura Ulbrich, Christoph Kröger

**Affiliations:** 1 Department of Psychology, University of Hildesheim, Hildesheim, Germany; Philipps-University of Marburg, Marburg, Germany

**Keywords:** depressive disorders, quality-adjusted life years, willingness to pay, quality of life, electroconvulsive therapy

## Abstract

**Background:**

As estimated by the World Health Organization, depressive disorders will be the leading contributor to the Global Burden of Disease by 2030. In light of this fact, we designed a study whose aim was to investigate whether the value placed on health-related quality of life (HRQoL) for a depressive disorder is higher in patients diagnosed with a major depressive disorder (MDD) compared to non-patients in a matched sample.

**Method:**

We collected data on willingness to pay (WTP) for a total of four health-gain scenarios, which were presented to 18 outpatients diagnosed with a MDD versus 18 matched non-patient respondents with no symptoms of depression. Matching characteristics included age, income, level of education, and type of health insurance. Respondents were presented with different HRQoL scenarios in which they could choose to pay money to regain their initial health state through various treatment options (e.g., inpatient treatment, electroconvulsive therapy). To test whether the probability of stating a positive WTP differed significantly between the two samples, Fisher’s exact test was used. Differences regarding stated WTP between the samples were investigated using the Mann-Whitney U-test.

**Results:**

For most of the health scenarios, the probability of stating a positive WTP did not differ between the two samples. However, patient respondents declared WTP values up to 7.4 times higher than those stated by matched non-patient respondents.

**Conclusion:**

Although the perceived necessity to pay for mental-HRQoL gains did not differ between respondents with MDD and respondents with no symptoms of depression, patient respondents stated higher values.

## Highlights

- The probability of stating a positive WTP did not differ between samples.
- However, patient respondents stated WTP values as much as 7.4 times higher than non-patients.

The global burden of disease is shifting from premature death to years lived with disability (GBD 2017 DALYs and HALE Collaborators, 2018; Licher et al., 2019; Vigo et al., 2019). For this reason, the promotion of mental health has become a priority for health policies and action plans around the world (e.g., World Health Organization, 2013). Over the past several decades, the disease burden attributed to depressive disorders has increased tremendously, ranking them among the three leading causes of years lost due to disability (YLD; GBD 2016 DALYs and HALE Collaborators, 2017), as well as disability-adjusted life years (DALYs; Murray et al., 2012). By 2030, unipolar depression is estimated to be the leading factor within the global burden of disease (World Health Organization, 2008).

## Cost-Effectiveness Analyses

Due to limited resources in the health-care sector, cost-effectiveness analyses are used as guidelines in priority setting, resource allocation, and reimbursement decisions. The preferred metric of health benefits in cost-effectiveness analyses is commonly the measurement of quality-adjusted life years (QALYs), combining the impact of health benefits on both health-related quality of life and quantity of life years (Sund & Svensson, 2018). Additionally, this measurement facilitates the comparison of different interventions within a disease or in comparison with other diseases (Pennington et al., 2015). From a health–economic perspective, the preference for and value of health-care interventions can be assessed by estimating a person’s willingness to pay (WTP) for health gains (Sund & Svensson, 2018). The elicitation of preferences usually follows a two-stage process: 1) if the respondent indicates whether he or she is willing to pay money (yes/no); and 2) if the respondent indicates ‘yes’, that he or she is willing to pay money, the amount of money the respondent is willing to pay is further assessed.

## Willingness to Pay for a Quality-Adjusted Life Year

Various studies have tried to estimate the value of a QALY through the WTP method (e.g., Ahlert et al., 2013; Donaldson et al., 2011; Igarashi et al., 2019; Pennington et al., 2015). A systematic review including 24 studies on WTP per QALY found that WTP estimates range from €1,000 to €4,800,000, with mean WTP estimates of €118,839 and median estimates of €24,226 (Ryen & Svensson, 2015). Currently, preferences for health treatments are commonly elicited from the general public due to the recommendations of the Washington Panel on Cost-effectiveness in Health and Medicine (Gold et al., 1996) and the United Kingdom’s National Institute for Health and Care Excellence (National Institute for Health and Care Excellence, 2013). Recently, however, arguments for eliciting the preferences based on appraisals of persons suffering from the health condition in question have been discussed (for a systematic overview on these arguments, see Helgesson et al., 2020).

## Effects of Contextual and Individual Characteristics on WTP per QALY

WTP per QALY seems to be related to several *contextual* factors, such as duration (e.g., 0.1 QALYs over 10 years vs. 0.25 QALYs over 4 years), timing (i.e., QALY gain at the end of life vs. in the near future), and type of QALY gain valued (i.e., life extension vs. quality-of-life improvements), as well as the type and severity of the illness presented (Igarashi et al., 2019; Ryen & Svensson, 2015). Additionally, several *individual* characteristics seem to influence the stated values for health gains. The most common predictor effect was found for income: A higher household income significantly increased the probability to state a positive WTP (Ahlert et al., 2013), as well as increasing the amount of money respondents were willing to pay (Igarashi et al., 2019; Pennington et al., 2015). Also, individuals with a higher level of education stated greater amounts than individuals with fewer years of schooling (Ahlert et al., 2013; Pennington et al., 2015). The effect of age on WTP was significant in two large samples, but results showed inconsistent findings: While one study found that younger respondents stated higher amounts (Ahlert et al., 2013), Pennington and colleagues (2015) found a contrary effect. A study of the German general population investigated the effects of the German health care system^1^1Unlike other European countries, Germany has a universal health-care system with two types of health insurance: Germans can choose between public (statutory) insurance and private health insurance, which is co-financed by employer and employee. on WTP per QALY and found that respondents with private health insurance were willing to pay higher amounts for a QALY, even when controlling for income effects (Ahlert et al., 2013).

To the best of our knowledge, no study has ever investigated the effects of the individual relevance of the presented health-gain scenario on the respondent’s WTP per QALY. Additionally, several studies argued that the plurality of different perspectives should be acknowledged, and that values for health benefits (i.e., QALYs) should be based on preferences from both patients and the general public (Dolan, 2009; Ogorevc et al., 2019; Versteegh & Brouwer, 2016). A meta-analysis assessed whether values for QALYs differed between patients and the general public, comparing different valuation methods (time trade-off, visual analogue scale and standard gamble; Peeters & Stiggelbout, 2010). However, preferences from patients and the general public using the WTP method have yet to be investigated.

## Study Aims

With an eye toward this need for more specific information on patient and non-patient preferences, the aim of our study was to assess whether WTP preferences for mental health gains differ between outpatients with a diagnosed major depressive disorder (the patient sample) and respondents from the general public with no symptoms of depression (the non-patient sample). To control for the effects of the above-mentioned individual characteristics on WTP, we matched respondents from the patient sample with respondents from the non-patient sample based on income, level of education, age, and type of health insurance (see Section ‘Participants and Procedures’). The above-mentioned meta-analytical comparison of patient and non-patient health-state assessments found that patients give higher valuations than non-patients (Peeters & Stiggelbout, 2010). Therefore, we aim to investigate the following hypotheses:

1. The probability of indicating a positive WTP (WTP > 0) is higher throughout all the scenarios in the patient sample compared to its likelihood among respondents with no self-reported symptoms of depression (the non-patient sample).
2. Respondents from the patient sample are willing to pay significantly higher amounts for the health gains presented than respondents with no self-reported symptoms of depression (the non-patient sample).

## Method

### Ethics Approval

This study was performed in accordance with the principles of the Declaration of Helsinki. The Ethical Review Committee of the University of Hildesheim, Germany, approved the study (Application number: 107).

### Participants and Procedure

#### Patient Sample

Individuals with a suspected depressive disorder were screened at a German university outpatient clinic between May 2019 and March 2020. Possible participants were informed as to the objective of the study both verbally and in writing, and were required to provide their written consent. Participants were eligible for inclusion if they were more than 18 years of age and met the *DSM-5* criteria of a major depressive disorder, using the German version of the Structured Clinical Interview for the *Diagnostic and Statistical Manual of Mental Disorders*, fifth edition (*DSM-5*), Clinical Version (SCID-5-CV; Beesdo–Baum et al., 2019). One master-level psychologist and three bachelor-level research-assistants conducted the interviews. All four interviewers had been trained in the administration and scoring of the SCID-5-CV in a workshop conducted by the second author, who is a licensed interviewer. The ratings of the diagnoses in question were discussed with the attending psychotherapist. We excluded patients who showed indications of mental retardation or dementia, substance-dependence disorders, bipolar disorder, or schizophrenia. Patients with other co-occurring mental disorders were not excluded. After the SCID-5-CV interview, patients who met the inclusion criteria and consented to participating in the study were asked to answer the questions of the online survey (further described in Section ‘Online Questionnaire’) on a laptop that we provided. After completing the survey, patients were thanked for their participation in the study.

#### Non-Patient Sample

For each respondent in the patient sample, we compared one matched respondent from the German general population who reported no symptoms of depression. Computer-based matching was conducted using the following characteristics: age at index rate (± 8 years), income category (see Table 2), highest level of education (basic, secondary, or advanced), and type of health insurance (statutory vs. private). Respondents from the German general population were recruited from an Internet panel run by an independent research institute (USUMA GmbH; http://www.usuma.com/) between March 6, 2019 and March 25, 2019. The research institute we selected complied with the ESOMAR International Code on Market, Opinion, and Social Research and Data Analytics. Internet-panel participants were informed about the online survey via email. After completing the survey, participants received survey ‘reward’ points from the Internet-panel company, which they could exchange for an online gift certificate or merchandise.

### Online Questionnaire

On the first page of the online questionnaire, respondents were informed about the objective of the study and were asked to give their consent. The hypothetical scenario that was introduced assumed that no sickness funds exist in Germany, and therefore, respondents would not have to pay premiums or contributions toward health insurance, increasing their monthly net income by that amount. Respondents were asked to imagine that instead, they would need to pay for every medical service out of their own pocket.

The concept of measuring health on a visual analog scale was introduced: Based on the European Quality of Life 5-Dimensions 3-Level Version (EQ-5D-3L; Szende et al., 2007), three health states and numerical valuations derived from survey values (Dolan et al., 1999) were used to indicate different levels of health on the scale. Demographical questions (e.g., age, income, health insurance, pre-existing diseases, region of residence) were presented. Respondents were then asked to estimate their life expectancy, and to rate the current state of their health on the European Quality of Life Visual Analogue Scale (EQ-VAS; Szende et al., 2007), with values between 0 and 100. Using items of the Patient-Health Questionnaire (PHQ-2; Kroenke et al., 2003) and EQ-5D-3L (Szende et al., 2007), respondents were asked to briefly assess their symptoms of depression and current health-related quality of life. The PHQ-2 is a two-item, self-administered depression module that scores the two main criteria from the *DSM-5*. Answer categories range from 0 (“not at all”) to 3 (“nearly every day”), and the total severity score ranges from 0 to 6. Regarding the total value of the PHQ-2 in the patient sample, the internal consistency was good (α = .82). A cut-off score of ≥ 3 (see Kroenke et al., 2007) proved to be most suitable regarding sensitivity and specificity for the diagnosis of a major depressive disorder.

Next, a description of typical symptoms of depressive disorders and their impact on everyday life, including mortality rates by suicide, was presented (see Online Resource 1 in the Supplementary Materials). The respondents were given four different scenarios of health loss of either one QALY (Scenarios A and B) or a fraction of a QALY (Scenarios C and D), due to a depressive episode. These scenarios, which are further described in Table 1, were presented in random order. The order of the questions and the wording of one sample scenario are displayed in Online Resource 2 (see Supplementary Materials). The respondents were asked if they were willing to pay money for each of the presented health-gain scenarios. If the respondents answered “yes,” that they would be willing to pay money for treatment, a table with three columns was presented, with a series of values in Euros ranging from €10 to €300,000 in accordance with previous studies (Ahlert et al., 2013; Donaldson et al., 2011; Pennington et al., 2015). To facilitate decision-making, the respondents were asked to sort the Euro values into one of three columns, indicating which amounts they would be willing to pay, the amounts they would not be willing to pay, and the amounts that left them unsure about whether or not they would pay. In order to summarize the maximum amount that the respondent was willing to pay and the minimum that he or she was not willing to pay, the respondent was asked to state his or her maximum WTP as an open-ended response. If the respondent answered that he or she would not be willing to pay money for the presented health-gain scenario, several pre-coded responses (translated from the EuroVaQ study) and a free text option were presented. Lastly, respondents were asked to rate how much they currently knew about electroconvulsive therapy (ECT), which was offered as a treatment method in one of the scenarios^2^2This treatment method was used because its efficacy is recognized by the German Association for Psychiatry, Psychotherapy, and Psychosomatics (DGPPN), and because it is a highly standardized procedure with rapid response rates (DGPPN et al., 2015).. If they indicated that they knew at least “a little” about ECT, they were asked to state whether they thought this method was adequate. Respondents were given the chance to view and change their answers in the recapitulation section on the last page. The feasibility and validity of the questions were examined by pilot respondents who provided detailed feedback prior to the development of the survey.

**Table 1:** Health Gains Valued

Scenario	Health gain	Duration	Time	Initial health state achieved?	Treatment
A	25 points	4 years	In 1 year	100%	pain-free treatment
B	10 points	10 years	In 1 year	100%	pain-free treatment
C	25 points	4 years	In 1 year	90%	8-week inpatient treatment
D	25 points	4 years	In 1 year	90%	8-week inpatient treatment plus electroconvulsive therapy

### Exclusion Criteria

To ensure that the questions were relevant to the individual respondents, and in accordance with the EuroVaQ report (Donaldson et al., 2011), the following exclusion criteria were applied:

#### General Exclusion Criteria

Respondents who indicated that “the government should pay” from the set of pre-coded responses as the reason for zero WTP (so-called “protest respondents”), were excluded due to their not having understood the hypothetical nature of the scenario (as is standard for WTP studies; see Olsen & Donaldson, 1998; Pennington et al., 2015).

#### Scenario-Specific Exclusion Criteria

Additionally, respondents were excluded from data analysis regarding scenarios A, C, and D if they rated their health state at less than 35 points (indicating poor health), and if they expected to live for less than 6 years as of that day. Respondents were excluded from data analysis regarding scenario B if they rated their health state at less than 20 points, and if their life expectancy was assumed to be below 12 years. The intention was to ensure that no health loss reduced the respondent’s health to below 10 points, and that all health gains were complete at least one year before the respondent expected to die.

### Data Analysis

All analysis was undertaken with IBM SPSS Statistics 26. The collection of open-ended responses allowed us to determine the mean and median values reported for each scenario, which were collected in Euros. The current study does not report trimmed means because Ahlert and colleagues (2013) found that trimming the top 1% or 5% of WTP values may lead to the exclusion of potentially reasonable cases (e.g., younger respondents with a higher income). The Kolmogorov–Smirnoff Test and Q-Q plots indicated that the assumption of normal distribution was violated: Distribution of WTP scores for scenario A (*D*(20) = 0.385, *p* < .001), scenario B (*D*(20) = 0.416, *p* < .001), scenario C (*D*(20) = 0.363, *p* < .001), and scenario D (*D*(20) = 0.270, *p* < .001) all differed significantly from normal.

To test Hypothesis 1 — whether the likelihood of expressing a positive WTP differed across both samples — WTP responses were dichotomized as zero and non-zero values. Because of the small sample size, Fisher’s exact test and odds ratios were calculated. To assess Hypothesis 2 — whether WTP values for the described health gains differed between the patient and the non-patient sample — the nonparametric Mann–Whitney *U*-Test was applied, due to the skewed distribution of the WTP scores. Effect size *r* was calculated by dividing the *z*-scores for the test statistic by the square root of the sample size (Field, 2018; Rosenthal, 1991). Bias-corrected accelerated 95% confidence intervals around means were estimated.

## Results

### Sociodemographic Characteristics of the Samples

**Figure 1:**
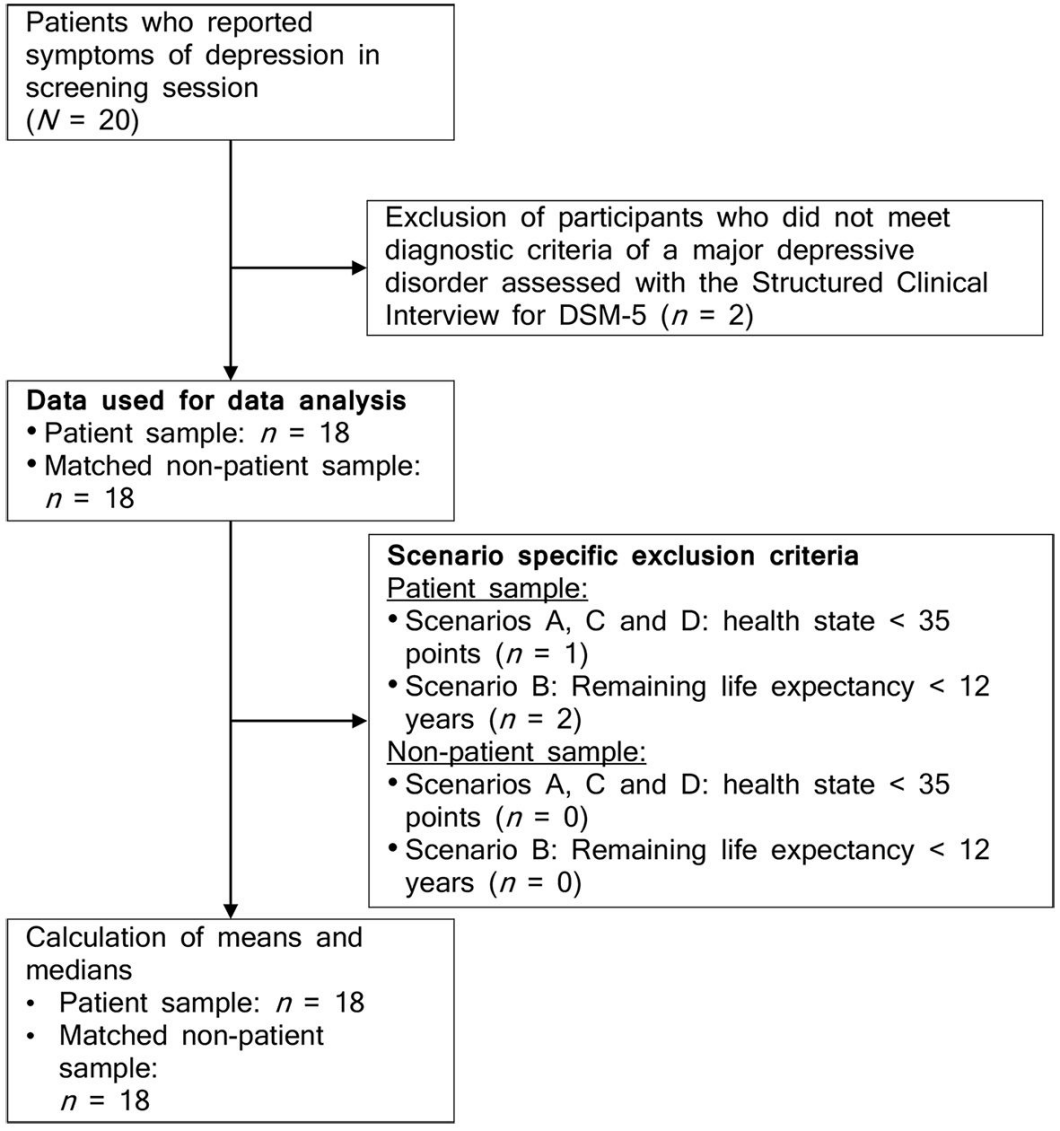
Flowchart

Figure 1 depicts the flowchart. A total of *N* = 36 participants were included in the study, with *n* = 18 participants in each sample. Most of the total sample (75%) was female, with a mean age of 48 years (*SD* = 14.88).

#### Patient Sample

From an initial sample of 20 screened outpatients, *n* = 18 patients met the *DSM-5* criteria of a major depressive disorder. No co-occurring mental disorders were diagnosed. The cut-off score of the PHQ-2 was exceeded by 16 patients (88.9%), while the mean score was 4.33 (*SD* = 1.57). The mean overall health state of the patient sample was indicated as poor (*M* = 61.67; *SD* = 18.31).

#### Matched Non-Patient Sample

The matching process based on income, level of education, type of health insurance, and age resulted in a sample of *n* = 18 matched respondents from the German general population. We ensured that respondents of the matched non-patient sample reported no symptoms of depression (PHQ-2 sum score = 0). The mean overall health state of the non-patient sample was indicated as rather good (*M* = 89.94, *SD* = 9.17).

Table 2 displays the sociodemographic characteristics of the two samples. No between-group differences were found in terms of the sociodemographic data. None of our subjects had to be excluded as protest respondents.

**Table 2:** Sociodemographic Characteristics of Both Samples

Characteristic	Patient Sample *N* = 18		Non-patient sample *N* = 18	
	*M* (*SD*)	Min/Max	*M* (*SD*)	Min/Max
Age (in years)	48.33 (15.22)	22/77	47.89 (14.97)	22/70
Life expectancy (age)	82.28 (9.49)	65/99	83.78 (8.45)	70/110
Health status (0-100)	61.67 (18.31)	20/95	89.94 (9.17)	70/100
	*n*	%	*n*	%
20 to 69 (poor)	11	61.1	0	0.0
70 to 79 (rather poor)	2	11.1	2	11.1
80 to 89 (rather good)	4	22.2	2	11.1
90 to 100 (very good)	1	5.6	14	77.8
Low remaining lifetime (< 16 years)	4	22.2	1	5.6
Females (rather than males)	16	88.9	11	61.1
Educational level				
Basic (nine years)	0	0.0	0	0.0
Secondary (ten years)	8	44.4	7	38.9
Tertiary (> ten years)	10	55.6	11	61.1
Monthly household income				
No answer	1	5.6	1	5.6
Below 500 €	0	0.0	0	0.0
500 to below 1.000 €	1	5.6	1	5.6
1.000 € to below 1.500€	1	5.6	1	5.6
1.500€ to below 2.000€	4	22.2	4	22.2
2.000€ to below 3.000€	4	22.2	4	22.2
3.000€ to below 4.000€	6	33.3	6	33.3
4.000€ and more	1	5.6	1	5.6
Health Insurance				
Social insurance	17	94.4	17	94.4
Private insurance	1	5.6	1	5.6
ICD-10 Diagnosis				
Depressive episode	8	44.4		
Recurrent MDD	10	55.6		

*Note. M* = mean; *SD* = standard deviation; Min/Max = Minimum/Maximum; *N* = sample size; MDD = major depressive disorder.

### Results Regarding Hypothesis 1: Probability of Indicating a Positive WTP

Results from Fisher’s exact test indicate no association between the sample (patient vs. non-patient sample) and the probability of stating a positive WTP (WTP > 0) in three of four scenarios (Scenarios B, C, and D). Only in scenario A was the probability of expressing a positive WTP higher in the patient sample compared to the non-patient sample (χ^2^ = 6.84, *p* < .05). Odds ratios could not be calculated, as 100% of the patient sample indicated a positive WTP.

In the patient sample, the number-one reason for being unwilling to pay for the presented health gains across all scenarios was: “The effects of treatment are too small.” In the non-patient sample, the number-one reason stated was: “It would not be so bad/I could live with it.” Table 3 shows the frequency of reasons stated for zero WTP.

**Table 3:** Frequencies of Reasons for Zero WTP

Scenario	*N* Zero WTP	It would not be so bad/ I could live with it	Effects of treatment are too small	I want my family to have the money	I would get better without treatment	I value the treatment but cannot afford it	Other reasons
Patient sample							
A	0	0	0	0	0	0	0
B	1	0	1 (6.3)	0	0	0	0
C	2	0	0	0	0	0	2 (11.8)
D	8	0	2 (11.8)	0	0	0	6 (35.4)
Non-patient sample							
A	6	1 (5.6)	0	1 (5.6)	1 (5.6)	1 (5.6)	2 (11.2)
B	7	4 (22.2)	0	1 (5.6)	0	1 (5.6)	1 (5.6)
C	6	1 (5.6)	0	0	2 (11.2)	2 (11.2)	1 (5.6)
D	8	2 (11.2)	0	0	2 (11.2)	2 (11.2)	2 (11.2)

*Note.* Percentages are in parentheses. *N* = sample size; WTP = willingness to pay.

### Results Regarding Hypothesis 2: WTP Differences Between Patient and Non-Patient Respondents

Mean, median, and maximum WTP values, as well as bias-corrected accelerated 95% confidence intervals around means, are displayed in Table 4. In the patient sample, mean WTP values ranged from €15,778 (Scenario D) to €54,794 (Scenario A). In the matched non-patient sample, mean values ranged from €2,277 (Scenario B) to €4,650 (Scenario A). Results from the Mann–Whitney *U*-Test indicated that patient respondents stated significantly higher WTP values than non-patients in all scenarios: Scenario A (*U* = 33.50, *z* = –3.05, *p* < .01, *r* = –.56), scenario B (*U* = 25.50, *z* = –2.97, *p* < .01, *r* = –.58), scenario C (*U* = 22.50, *z* = –3.31, *p* < .001, *r* = –.64) and scenario D (*U* = 10.50, *z* = –2.83, *p* < .01, *r* = .65). For all scenarios, differences between samples represented a medium effect in accordance with Cohen (1988).

**Table 4:** Mean, Median and Maximum Values in Euros (€) for Both Samples After Applying General and Scenario-Specific Exclusion Criteria

Scenario	*n_a_*	*n* WTP > 0	*M*	Bootstrapped 95% CI	*Mdn*	Maximum WTP
Patient sample						
A	17	17	54,794	14,646-116,424	15,000	350,000
B	16	15	52,667	6,956-121,249	10,000	350,000
C	17	15	23,867	10,714-45,548	10,000	150,000
D	17	9	15,778	7,667-25,762	13,000	50,000
Non-patient sample						
A	18	12	4,650	2,322-7,686	2,500	15,000
B	18	11	2,277	1,000-4,126	1,500	10,000
C	18	12	3,433	2,245-4,737	2,750	10,000
D	18	10	2,415	1,183-3,567	1,750	5,000

*Note. n*_a_= sample size after applying scenario-specific exclusion criteria; *n* = sample size; CI = confidence interval.

## Discussion

### A Vital Assessment of Patient Preferences

As currently discussed (e.g., Dolan, 2009; Ogorevc et al., 2019; Versteegh & Brouwer, 2016), the present study is one of the first attempts to directly compare experience-based preferences from patients to ‘hypothetical’ preferences of the general population using the WTP method.

Results indicate that the probability of stating a positive WTP does not differ between patients and non-patient respondents. However, when assessing the number-one reasons indicated for zero WTP (patient sample: “Effects of treatment are too small,” vs. non-patient sample: “It would not be too bad/I could live with it”), it seems that respondents with no prior experience of depression underestimate the burden of depressive symptoms. As discussed by Dolan (2007), “hypothetical” preferences of the general public, as elicited through assessing WTP values, may not be a reliable basis for judgment because the “general public are not good at assessing what it would be like to experience different states of health” (Dolan, 2007, p. 6). However, contrary to the assumption that “hypothetical” preferences by the public tend to overestimate the severity of a loss of health (Dolan, 2007, p. 6), patients stated significantly higher WTP values than non-patients. These findings are in accordance with previous studies (Ogorevc et al., 2019; Versteegh & Brouwer, 2016) and emphasize the need to consider both the perspectives of the general public and those of patients when assessing values or preferences for health benefits.

In this study, we assessed respondents’ WTP for one specified treatment (electroconvulsive therapy) in detail due to its high standardization when compared to other psychotherapeutic interventions. Thus, when assessing results for this specified scenario, it seems unexpected that only 53% of the patient sample and 55% of the non-patient sample were willing to pay money for ECT. One possible explanation might be that 83% of the patient sample stated that they knew nothing or little about ECT, compared to 72% of the non-patient sample. The present findings accord with the conclusion of a recent study, which found that ECT is still largely underutilized due to persisting stigma and lack of knowledge about modern ECT techniques (Kellner et al., 2020). In particular, considering recent discussions of advocating for patients in the decision-making process regarding treatment options (e.g., Barry, 2011; Couët et al., 2015), the present findings underline the importance of an informed patient. So-called patient-decision aids — tools designed to help patients make an informed choice, which include explanations about treatment options based on scientific evidence — can be used to improve patients’ knowledge of which treatment route to choose, as well as the risks and benefits of various treatments (for an overview, see Perestelo‐Perez et al., 2017).

The cost-effectiveness of primary care for depressive disorders has been investigated by, for example, Chisholm et al. (2004) and Pyne et al. (2003). Low-cost, non-medical interventions for relief from depression, such as exercise, relaxation, and bibliotherapy, are also readily available (for a systematic review, readers are referred to Morgan & Jorm, 2008). Their (cost-)effectiveness in reducing symptoms of depression is, however, yet to be assessed in randomized controlled trials in a clinical population (Bellón et al., 2021; Lawlor & Hopker, 2001; Philippot et al., 2019)

### Strengths and Limitations

Matching the respondents from the patient sample to respondents from the non-patient sample allowed us to control for the effects of individual characteristics (e.g., income, level of education) on WTP. Presenting the scenarios in a randomized order let us control for ordering effects. However, some limitations should be also mentioned.

First, the size of both samples (*n* = 18 in each sample) was quite small, and the post-hoc power analysis indicated medium power (1– β = 0.89), assuming a medium effect size (|*d*| = 0.5, according to the convention of Cohen (1988)).

Second, the broad majority (88.9%) of the recruited patient sample was female. Results from the EuroVaQ study indicate that men stated a higher WTP (Donaldson et al., 2011, p. 76). Still, Ahlert and colleagues (2013) investigated the effect of gender in more detail, and found that although women were significantly more likely than men to state a positive WTP, males were willing to pay significantly higher amounts than females. Therefore, generalization of results may be limited, and a more representative patient sample should be recruited in subsequent studies.

Additionally, presenting scenarios that emphasize the certainty of successful treatment — which may be especially unlikely with respect to mental health — may have led to the overestimation of estimated WTP values. More scenarios with uncertainty characteristics should be evaluated in further research, as well as other specified treatment options, such as psychotherapeutic treatment approaches or antidepressant medication (DGPPN et al., 2015).

Fourth, the assessment of the variable “knowledge about ECT” consisted of one item only, and did not objectively specify how much respondents know or how and where they became informed (e.g., movies, media, medical services). During administration of the present survey, a measure to assess perceptions and knowledge of ECT was published (Tsai et al., 2020), and we believe that it should be used in future studies to guarantee an objective, more detailed measurement of the respondents’ attitudes toward and knowledge of ECT.

Additionally, we only recruited people who were being seen at an outpatient clinic. It is possible that patients of an inpatient clinic with more severe depressive symptoms would place higher values on mental-health-related quality of life, and might also be better informed about their treatment options — ECT in particular. Generalization of results may therefore be limited to patients from an outpatient setting with no co-occurring mental disorders.

Finally, the health-care system (including psychiatric and psychological care) in Germany is unique compared to that of other European systems (see Melcop et al., 2019, for an overview). In Germany, health insurance is mandatory, and Germans can choose between public or private health insurance. Access to mental health care is free of additional charges in Germany, which is uncommon among the other European Union member states (Strauß, 2009). Additionally, the mental-health-care spending proportionate to the gross domestic product is higher in Germany (4.8%) than the European average (4.1%), and is only exceeded by that of Denmark (5.4%; OECD, 2018). Therefore, external validity may be limited to countries with similar health services for mental disorders.

## Conclusion

This study investigated the effect of the personal relevance of a presented health-gain scenario on the respondent’s WTP per QALY, and produced findings that add valuable information toward estimating the effects that individual characteristics have on the value that respondents place on a QALY. Additionally, our findings emphasize the need to assess hypothetical population preferences alongside actual patients’ preferences for health benefits.

## Supplementary Materials

The Supplementary Materials contain the following items (for access see Index of Supplementary Materials below):

Supplementary Material 1: Translation of the health state description
Supplementary Material 2: Sample scenario

10.23668/psycharchives.5286 Supplement 1 Supplementary materials to "Monetary valuation of a Quality-AdjustedLife Year (QALY) for depressive disorders among patients and non-patient respondents: A matched willingness to pay study"

UlbrichL.
KrögerC.
 (2021). Supplementary materials to "Monetary valuation of a Quality-Adjusted Life Year (QALY) for depressive disorders among patients and non-patient respondents: A matched willingness to pay study"
[Additional information]. PsychOpen. 10.23668/psycharchives.5286
PMC966722236398289
